# Electroconvulsive therapy in a tertiary Australian mental health facility between 2009 and 2020

**DOI:** 10.1177/00048674241256839

**Published:** 2024-06-03

**Authors:** Emily Martin, Subramanian Purushothaman, Emma Ballard, Julie A Blake, Kylie Burke, James G Scott

**Affiliations:** 1 Metro North Mental Health Service, Metro North Hospital and Health Service, Herston, QLD, Australia; 2 Brain and Mental Health, QIMR Berghofer Medical Research Institute, Herston, QLD, Australia; 3 Child Health Research Centre, The University of Queensland, South Brisbane, QLD, Australia; 4 Child and Youth Mental Health Research, Queensland Centre for Mental Health Research, Wacol, QLD, Australia; 5 Child and Youth Mental Health Service, Queensland Children’s Hospital, South Brisbane, QLD, Australia; 6 School of Psychology, The University of Queensland, South Brisbane, QLD, Australia; 7 ARC Centre of Excellence for Children and Families Over the Life Course, Institute for Social Science Research, The University of Queensland, South Brisbane, QLD, Australia

**Keywords:** Mental disorders, schizophrenia, depression, neurostimulation

## Abstract

**Background:**

Despite electroconvulsive therapy being one of the most effective treatments in psychiatry, few studies report trends in the provision of electroconvulsive therapy over time. This study aims to investigate the use of electroconvulsive therapy between 2009 and 2020 in an Australian public tertiary mental health facility, and to describe the electroconvulsive therapy patient population and change in courses of treatment.

**Methods:**

Routinely collected data for 677 patients who received 1669 electroconvulsive therapy courses of treatment at an Australian public tertiary mental health facility between 2009 and 2020 were examined.

**Results:**

The provision of acute electroconvulsive therapy was stable across the study period; however, the number of maintenance electroconvulsive therapy courses commenced declined over the study. Schizophrenia was the most common indication for index treatment (37.4%). The majority of patients (85.7%) received acute electroconvulsive therapy only. Voluntary provision of electroconvulsive therapy declined over the study period, reducing from 44.9% in 2009 to 16.3% in 2020.

**Conclusion:**

Over the study period, there was a significant reduction in the number of maintenance electroconvulsive therapy courses commenced, and a large increase in involuntary treatment. The provision of electroconvulsive therapy was more likely to occur in males with a diagnosis of schizophrenia. Further studies are needed to generate a greater understanding of the factors influencing the provision of electroconvulsive therapy within differing geographical, social and healthcare landscapes.

## Introduction

Electroconvulsive therapy (ECT) is a safe and effective treatment for the management of affective, psychotic and neuropsychiatric disorders (Galletly et al., 2016; Malhi et al., 2015). There has been increased demand for admissions to mental health services within Australia in recent years (Royal Commission into Victoria’s Mental Health System, 2019; Zhang et al., 2011) with a corresponding need to reduce the length of stay for acute admissions. Pharmacological advancements such as clozapine which reduces length of admissions (Leucht et al., 2013; Lin et al., 2020; Siskind et al., 2019) and long-acting injectable formulations of antipsychotic medications which prevent relapse of psychosis (Pacchiarotti et al., 2019) have changed the use of ECT. The stigma around ECT reduces its acceptability as a treatment by both those with mental illness and clinicians (Griffiths and O’Neill-Kerr, 2019; Ithman et al., 2018; Kring et al., 2018; Matthews et al., 2016; Surya et al., 2015) despite the fact that it is recognised as an effective treatment for acute psychosis as well as affective disorders (Elias et al., 2021; Hermida et al., 2018). ECT is resource intensive requiring general anaesthetic which can impede access to this treatment (National Institute for Health and Clinical Excellence, 2009). Advances in knowledge about anaesthetics and methods of ECT delivery have reduced the associated cognitive impairment, making it safer and potentially improving its acceptability (Gazdag and Ungvari, 2019; Griffiths and O’Neill-Kerr, 2019). There is robust evidence that a significant minority of people with major depressive disorder, bipolar disorder and schizophrenia have treatment refractory illnesses that only respond to ECT (Lally et al., 2016; Rosenquist et al., 2018).

In Australia, ECT is a regulated treatment under jurisdictional mental health acts (MHAs) with various provisions for the collection of data relating to its use. There are three studies that have examined trends in ECT provision in Australia over time. Teh et al. (2005) examined trends in Western Australia using data from the state mental health information system and psychiatric hospital records between 1997 and 2001. The authors found that rates of treatment with ECT had almost doubled over the study period. Doessel et al. (2006) utilised Medicare data to examine private ECT provision in Australia between 1984 and 2004 and found that after an initial decline, there was a steady increase in the use of ECT. The authors suggested this increase was due to improvements in ECT monitoring and uptake of right unilateral treatment which contributed to increased acceptability and use. Plakiotis et al. (2012) examined the provision of ECT in Victoria between 1998 and 2007 and found that ECT rates decreased overall. The study found ECT to be predominantly delivered to female patients (62%), to those with a depressive disorder (80%), and that unilateral placement was more common in depressive illnesses and bilateral in psychotic illnesses. Improvements in pharmacological treatments were suggested as an explanation for the overall decline in ECT rates. In summary, there are limited Australian studies and of those that have been conducted, only state or federally based registry data are used and the data are now outdated. Moreover, during the timeframes in which the studies were conducted, there was no consistent trend in the use of ECT over time.

Internationally, the trends in ECT use have been reported in five studies. Studies of registry data from Denmark (Munk-Olsen et al., 2006), Portugal (Mota et al., 2021) and Hong Kong (Chung, 2003) have reported a stable pattern of ECT use over time. In a single hospital in China, ECT increased between 2014 and 2017 to 17.8% of patients receiving this treatment (Ma et al., 2019). Relative affordability, reduced stigma and broader treatment applications extending to include agitation and aggression and its more frequent use in the adolescent population were suggested factors influencing greater use in this study. By contrast, Buley et al. (2017) examined the use of ECT in the United Kingdom over two discrete periods: 2012–2013 and 2014–2015 and compared these with previously collected surveys in 2006. ECT rates declined both in number of courses and number of clinics providing ECT. Of note, they found an increase in the proportion of patients receiving ECT under the MHA suggesting it was being predominantly used for treating individuals with severe illness. In summary, there are different trends in the provision of ECT internationally.

Despite ECT being one of the most effective treatments in psychiatry, controversy about its use continues (Read et al., 2019). For this reason, it is surprising there are few studies reporting on the trends in the provision of ECT over time. Of the studies that do exist, they use registry-based data, which provide limited clinical information about the patients receiving treatments. Very few services have collected and published detailed data on ECT usage over an extended timeframe. The primary aim of this study is to examine the use of ECT treatment between 2009 and 2020 in an Australian public tertiary mental health facility. The secondary aims of this study are to describe the ECT patient population and changes in courses of treatment.

## Methods

### The ECT service

The Royal Brisbane and Women’s Hospital (RBWH) is a public tertiary teaching hospital with 91 inpatient psychiatric beds including a 12-bed adolescent unit, 10-bed old age psychiatry unit and 6-bed eating disorder unit. Staffing for the ECT service comprises an ECT director, ECT coordinator and Assistant in Nursing. The anaesthetic team includes a consultant anaesthetist and an anaesthetic technician. Psychiatry registrars attend on three monthly rotations to provide clinical support and for their professional development. ECT treatments are conducted 3 days per week. A dedicated ECT suite is adjacent to one of the inpatient units and has a treatment room and a recovery room. The recovery area includes two dedicated nurses to assist patients in recovery.

The ECT suite has capacity for up to 12 treatments per session and is equipped with a Thymatron System IV (Somatics LLC, Venice, Florida, USA). Anaesthesia for ECT involves a short general anaesthetic and the agent used is predominantly propofol with thiopentone on some occasions. Suxamethonium is the muscle relaxant used, although other muscle relaxants are available if required. Patients are generally admitted as inpatients and have three weekly ECT treatments; however, outpatient ECT treatment can be facilitated provided there is a responsible adult to provide 24 hours of patient supervision, and patients are aware and compliant with the fasting requirements before ECT.

### Data collection

The Office of the Chief Psychiatrist in Queensland requires the collection of data on the provision of ECT on a 6-month basis. In response to this requirement, the RBWH developed a Microsoft Access-based database, which has been continuously maintained since 2008. This was to provide a robust, organisation-wide system of reporting that ensures and informs patient care as per National Safety and Quality Health Service Standards 5.1 (Australian Commission on Safety and Quality in Health Care, 2017). Data are entered electronically by either the treating psychiatrist or ECT nurse following each treatment session and are confirmed and checked by the anaesthetist, psychiatrist and psychiatry registrar involved in the ECT provision on that day. The database captures the number of treatments, type of consent, electrode position, dose, motor and electoencephalogram (EEG) seizure duration, anaesthetic medications used and their doses. Treatment categories include acute, continuation and maintenance.

### Legislation governing the use of ECT in Queensland

In Queensland, the MHA governs the treatment and care of individuals within the health system who have mental health conditions. Throughout the study, ECT was a regulated treatment that could only be legally provided under the MHA. The MHA 2000 was replaced by the MHA 2016 on 01 March 2016. Under both MHAs (2000 and 2016), voluntary ECT can be administered when clinically indicated to a patient who has capacity and provides informed consent to the treatment. When ECT is clinically indicated and the patient does not have capacity and/or does not consent, ECT can only be provided with the approval of a Mental Health Review Tribunal (MHRT). The MHRT is an independent panel consisting of a lawyer, a psychiatrist and a community member who is not a psychiatrist or lawyer. The tribunal approves or declines the provision of ECT, determined by information provided by the treating team, the patient and their nominated support person. With the introduction of the MHA 2016, the MHRT has been required to appoint a lawyer to represent the patient at no cost to the individual in hearings for an involuntary ECT application.

ECT may be performed on an involuntary patient in emergency circumstances without prior approval of the tribunal if a psychiatrist has made a treatment application to the tribunal; and the psychiatrist and the medical superintendent at the mental health service where the treatment is to be given have certified in writing that it is necessary to perform emergency ECT to save the patient’s life, or prevent the patient from suffering irreparable harm (MHA 2000, MHA 2016).

### Study definitions

A course of *acute* ECT is provided to patients experiencing an episode of illness and would generally consist of a maximum of 12 treatments delivered between 2 and 3 treatments per week. A course of acute ECT could extend beyond 12 treatments if the patient was clinically improving but still unwell. *Maintenance* ECT is delivered to patients following a course of acute ECT who require ongoing ECT treatment to prevent illness relapse. Maintenance ECT is delivered as a course of up to 12 treatments, usually between once a week to once every 2 or 3 weeks. *Inpatient* ECT refers to ECT provided to individuals receiving treatment in hospital. *Community* ECT refers to the provision of ECT to patients living in the community. They are usually admitted on the morning of the treatment and then discharged the same day following recovery. *Primary diagnoses* were grouped as schizophrenia, schizoaffective disorder, psychotic disorder, bipolar disorder or depression. Both bipolar mania and depression were included under the category of bipolar disorder due to small frequencies.

The decision to continue maintenance ECT after an acute course is based on the degree of clinical improvement, illness severity and treatment options for maintaining improvement. It is prescribed when it is the only effective treatment available to maintain symptom improvement and prevent relapse. Patients who receive maintenance ECT commonly have treatment-resistant psychosis and are taking clozapine, or treatment-resistant depression with recurrence of illness when only pharmacotherapy is provided.

Categorical variables are summarised as frequency and percentage and continuous variables as median and interquartile range (IQR). The dataset was analysed using SPSS version 25 (IBM Corp, 2017). This retrospective cohort study received ethics approval and a waiver of consent through RBWH Human Research Ethics Committee (HREC/2020/QRBW/72025). As the data presented in this paper cannot identify an individual, it does not fall within the definitions of ‘confidential’ or ‘personal’ or ‘health’ information under Queensland legislation.

## Results

A total of 677 patients received ECT during the period 2009 to 2020, 580 (85.7%) patients received acute ECT only, 10 (1.5%) received maintenance ECT only and 87 (12.9%) received both types of ECT. There were a total of 1669 courses administered with patients having a median of one (IQR 1–2, range 1–36) course of treatment.

Table 1 summarises demographics and treatment characteristics of patients at their index ECT treatment. Fifty-five per cent of patients were male with the largest group aged between 35 and 49 years (30.7%) at their index treatment. Schizophrenia was the most common indication (37.4%) followed by depression (33.8%), bipolar (13.3%) and schizoaffective disorder (11.4%), psychotic disorders (2.5%) and other disorders (1.6%). Treatment was voluntary for 29.4% of patients.

**Table 1.** Demographics, diagnosis and course of treatment for patients receiving electroconvulsive therapy at their index treatment between 2009 and 2020 (*n* = 677).

Demographics	*n* (%)
Gender (male)	372 (54.9)
Age (years)	
<18	19 (2.8)
18–24	70 (10.3)
25–29	65 (9.6)
30–34	64 (9.5)
35–49	208 (30.7)
50–64	156 (23.0)
65–74	43 (6.4)
≤75	52 (7.7)
**Diagnosis**	
Schizophrenia	253 (37.4)
Major depressive disorder	229 (33.8)
Bipolar disorder	90 (13.3)
Schizoaffective disorder	77 (11.4)
Psychotic disorder	17 (2.5)
Other	11 (1.6)
**Treatment**	
*Course*	
Acute	656 (96.9)
Maintenance	21 (3.1)
*Consent type*	
Emergency consent	160 (23.6)
MH review tribunal	318 (47.0)
Voluntary	199 (29.4)
*Course type*	
Community	33 (4.9)
Inpatient	644 (95.1)

MH: mental health.

**Figure 1.**
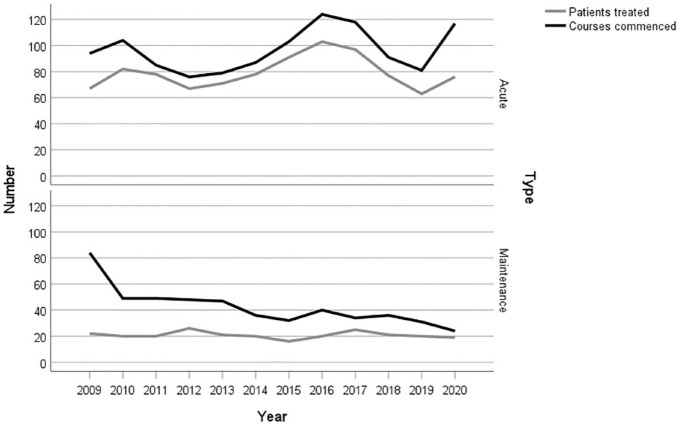
Trends in the number of patients and courses between 2009 and 2020 by acute and maintenance electroconvulsive therapy.

Figure 1 shows changes in patient numbers and courses for acute and maintenance ECT over time. The number of patients receiving acute ECT treatment each year ranged from 63 to 103 with no clear trend observed over time. The number of patients receiving maintenance ECT each year ranged from 19 to 26; however, the number of courses commenced each year decreased from 84 in 2009 to 24 in 2020. In 2009, all courses of maintenance ECT were categorised as commenced although, some of these patients would have in commenced their maintenance ECT in previous years. This would explain the very large reduction from 2009 to 2010. However, reductions in subsequent years are not a result of the way the maintenance ECT courses commenced was classified.

Table 2 summarises ECT therapy courses and patient diagnoses between 2009 and 2020. A total of 677 patients received 1669 courses of ECT comprising of 12,960 ECT treatments. In 2009, the year the study initiated, all patients were considered to be new to receiving ECT (*N* = 76). In subsequent years, the number of new patients reflected those who had never had ECT at the study hospital. The number of new patients and the total number of patients receiving ECT were very stable except for 2016 when both increased compared to the previous and subsequent years.

**Table 2.** Summary of ECT courses and patient diagnosis between 2009 and 2020.

Treatment	Overall	2009	2010	2011	2012	2013	2014	2015	2016	2017	2018	2019	2020
	*n* (%)	*n* (%)	*n* (%)	*n* (%)	*n* (%)	*n* (%)	*n* (%)	*n* (%)	*n* (%)	*n* (%)	*n* (%)	*n* (%)	*n* (%)
Number of new patients receiving ECT per year (*n*)	677	76	61	57	45	57	56	54	74	55	48	43	51
Number of patients receiving ECT per year (*n*)		76	93	89	85	89	90	103	114	109	87	75	88
Number of courses (*n*)	1669	178	153	134	124	126	123	135	164	152	127	112	141
Number of treatments (*n*)	12,960	1150	1128	1103	1134	980	981	1121	1266	1123	1021	883	1070
ECT category per course													
Acute	1159 (69.4)	94 (52.8)	104 (68.0)	85 (63.4)	76 (61.3)	79 (62.7)	87 (70.7)	103 (76.3)	124 (75.6)	118 (77.6)	91 (71.7)	81 (72.3)	117 (83.0)
Maintenance	510 (30.6)	84 (47.2)	49 (32.0)	49 (36.6)	48 (38.7)	47 (37.3)	36 (29.3)	32 (23.7)	40 (24.4)	34 (22.4)	36 (28.3)	31 (27.7)	24 (17.0)
Consent type per course													
Emergency consent	244 (14.6)	19 (10.7)	35 (22.9)	27 (20.1)	15 (12.1)	17 (13.5)	13 (10.6)	21 (15.6)	12 (7.3)	26 (17.1)	18 (14.2)	17 (15.2)	24 (17.0)
MH review tribunal	860 (51.5)	79 (44.4)	63 (41.2)	59 (44.0)	59 (47.6)	50 (39.7)	52 (42.3)	76 (56.3)	104 (63.4)	88 (57.9)	65 (51.2)	71 (63.4)	94 (66.7)
Voluntary	565 (33.9)	80 (44.9)	55 (35.9)	48 (35.8)	50 (40.3)	59 (46.8)	58 (47.2)	38 (28.1)	48 (29.3)	38 (25.0)	44 (34.6)	24 (21.4)	23 (16.3)
Course type													
Community	475 (28.5)	75 (42.1)	46 (30.1)	47 (35.1)	46 (37.1)	41 (32.5)	30 (24.4)	29 (21.5)	35 (21.3)	33 (21.7)	34 (26.8)	30 (26.8)	29 (20.6)
Inpatient	1194 (71.5)	103 (57.9)	107 (69.9)	87 (64.9)	78 (62.9)	85 (67.5)	93 (75.6)	106 (78.5)	129 (78.7)	119 (78.3)	93 (73.2)	82 (73.2)	112 (79.4)
Median number of courses per patient (median [IQR])	1 (1–2)	1 (1–4)	1 (1–2)	1 (1–2)	1 (1–2)	1 (1–2)	1 (1–2)	1 (1–2)	1 (1–2)	1 (1–2)	1 (1–2)	1 (1–2)	2 (1–2)
Diagnosis at commencement of course													
Schizophrenia	760 (45.5)	105 (59.0)	68 (44.4)	75 (56.0)	55 (44.4)	46 (36.5)	50 (40.7)	56 (41.5)	68 (41.5)	78 (51.3)	57 (44.9)	43 (38.4)	59 (41.8)
Schizoaffective disorder	207 (12.4)	32 (18.0)	32 (20.9)	16 (11.9)	15 (12.1)	16 (12.7)	9 (7.3)	9 (6.7)	10 (6.1)	19 (12.5)	15 (11.8)	14 (12.5)	20 (14.2)
Psychotic disorder	29 (1.7)	4 (2.2)	1 (0.7)	3 (2.2)	0 (0.0)	3 (2.4)	4 (3.3)	2 (1.5)	3 (1.8)	3 (2.0)	2 (1.6)	3 (2.7)	1 (0.7)
Bipolar	211 (12.6)	8 (4.5)	20 (13.1)	12 (9.0)	18 (14.5)	17 (13.5)	13 (10.6)	26 (19.3)	29 (17.7)	16 (10.5)	18 (14.2)	16 (14.3)	18 (12.8)
Depression	448 (26.8)	29 (16.3)	29 (19.0)	28 (20.9)	36 (29.0)	42 (33.3)	47 (38.2)	41 (30.4)	53 (32.3)	35 (23.0)	35 (27.6)	32 (28.6)	41 (29.1)
Other	14 (0.8)	0 (0.0)	3 (2.0)	0 (0.0)	0 (0.0)	2 (1.6)	0 (0.0)	1 (0.7)	1 (0.6)	1 (0.7)	0 (0.0)	4 (3.6)	2 (1.4)

MH: mental health; IQR: interquartile range; ECT: electroconvulsive therapy.

A total of 1159 (69.4%) acute and 510 (30.6%) maintenance ECT courses were administered between 2009 and 2020. The proportion of ECT delivered as acute courses increased over the study period, from 52.8% in 2009 to 83.0% in 2020. In contrast, maintenance courses decreased from 47.2% in 2009 to 17.0% in 2020. Inpatient provision of ECT was more common (71.5%) than outpatient (28.5%). Involuntary provision of ECT through the MHRT was the most common approval type (51.5%) and increased considerably over the study period, from 44.4% in 2009 to 66.7% in 2020. There was a large decrease in the proportion of courses of voluntary ECT from 47.2% in 2014 to 28.1% in 2015 which persisted for the remainder of the study. Schizophrenia was the most common indication for ECT (45.5%), followed by depression (26.8%).

The provision of ECT by diagnosis in acute and maintenance ECT is presented in Supplemental Tables 1 and 2, respectively. A total of 667 patients received an acute course of ECT during the study period and 97 received maintenance ECT. Of these 97, 87 had received an acute course of ECT at RBWH and 10 had been transferred to RBWH for maintenance ECT from other services. Both acute and maintenance ECTs were more likely to be provided to males for treatment of schizophrenia and females for affective disorders. Only 11.6% of patients with schizophrenia had voluntary acute ECT compared with 56.1% of patients with depression. The majority (83.0%) of maintenance ECT provided for depression was voluntary. The median number of acute ECT treatments was higher for people with schizophrenia (*n* = 10; IQR 6–16) compared with depression (*n* = 8; IQR 5–12).

## Discussion

This paper describes trends in ECT treatment from 2009 to 2020 in an Australian public tertiary mental health facility. Overall, ECT was most commonly used in the treatment of males with a diagnosis of schizophrenia. Depression was the second most common indication. There was high variation in the number of courses commenced and number of patients receiving acute ECT each year with no clear trend over the study duration. The number of courses commenced for maintenance ECT declined during the study period. In 2015, there was a very significant reduction in the proportion of ECT courses that were voluntary which persisted in subsequent years resulting in two-thirds of patients receiving involuntary ECT treatment in 2020.

Previous Australian studies report higher rates of ECT in females and in those with depression or affective psychosis illnesses (Plakiotis et al., 2012; Teh et al., 2005). More broadly, the international literature also reports higher rates of ECT use in females with affective illnesses (Buley et al., 2017; Chung, 2003; Ma et al., 2019; Mota et al., 2021; Munk-Olsen et al., 2006; Tor et al., 2019). These findings are likely due to the inclusion of both public and private hospital data. Tor et al. (2019), Mota et al. (2021) and Plakiotis et al. (2012) reported schizophrenia as the most common diagnosis for ECT in public hospital settings.

The relatively high rates of involuntary treatment at the RBWH are indicative of the patient population in public hospital settings. Involuntary mental health treatment is associated with severe illness, psychotic disorders, male gender, increased aggression and psychosocial problems (Canova Mosele et al., 2018; Hustoft et al., 2013; Kortrijk et al., 2010; Ng and Kelly, 2012). Both Ma et al. (2019) and Tor et al. (2019) suggested a relationship between severity of illness, ECT and involuntary status. A high proportion of patients receiving care in Australian tertiary public mental health services are males with schizophrenia who have treatment resistance often accompanied by aggression, suicidality and a lack of capacity to provide consent to treatment.

There was a reduction in the number of courses of maintenance ECT over the duration of the study, but not in the number of patients receiving them. We hypothesise this may be due to the availability of long-acting injectable antipsychotic medications. In 2010 and 2014, long-acting injectable paliperidone and aripiprazole, respectively, were approved by the Australian Therapeutic Goods Administration for acute and maintenance treatment of schizophrenia. Long-acting injectable antipsychotics are more effective than oral antipsychotics in preventing relapse of schizophrenia (Kishimoto et al., 2021).

There was a large increase in proportion of courses of ECT being involuntary from 2015. There was no notable change to the RBWH service or bed numbers over the duration of the study. In the absence of a clinical explanation for the change, the authors hypothesise that the increase in use of involuntary ECT may have occurred with the introduction of the 2016 MHA which was preceded by training and education of ensuring patients only received ECT if they were truly able to provide fully informed consent. Interestingly, the number of courses commenced and patients receiving acute ECT increased between 2014 and 2016, so the greater regulation of ECT in Queensland did not reduce the number of patients who received this treatment.

The socioeconomic demographic characteristics of the hospital service region must also be considered with regard to the higher rates of ECT provision in those with schizophrenia. During the study period, there were considerable changes to the population density of the community serviced by RBWH with an estimated extra 110,000 residents with the majority of this being in the inner city region (Coffee et al., 2016; Queensland Treasury, 2020). It has long been recognised that those with schizophrenia experience socioeconomic drift towards the central areas of a city (DeVerteuil et al., 2007). This potentially resulted in an increase in the illness severity and acuity of patients admitted to the RBWH over time which might account for some of the increase in the proportion of courses of involuntary ECT. However, the sudden rise in involuntary ECT from 2014 to 2015 is more likely to be explained by the implementation of legislative changes rather than a change to the patient population.

A major strength of this paper is the duration and breadth of clinical data collected on ECT, particularly when compared to previous studies that were of either shorter in duration or included fewer clinical indicators. Several limitations are also noted. Given the duration of the study period, various individuals have been responsible for data entry and interpretation relating to the database. As such, the data are vulnerable to both user error and bias as the person recording the data was also the provider of the treatment. Our study did not allow for overall comparison at the admission level between those who did and did not receive ECT at RBWH. A further limitation of this study was the diagnostic categorisation used which resulted in 65 primary diagnoses recorded being collapsed into six categories. The dataset does not allow for change of inpatient status during treatment limiting the ability to examine ECT rates among those accessing step-down facilities or transfer to the community prior to completion of the ECT course. The dataset also did not record individuals’ cultural backgrounds, which prevents any exploration of ECT provision to vulnerable minority groups.

## Conclusion

This paper has found that between 2009 and 2020 in an Australian tertiary public mental health service, there was no overall change in the number of courses, or number of patients receiving acute ECT; however, there was a reduction in the number of courses for maintenance ECT. ECT was more likely to be delivered to males aged between 18 and 35 with a diagnosis of schizophrenia, as an involuntary treatment. The strengthening of oversight of ECT that occurred with changes to Queensland MHA was the most likely explanation for an increase in the proportion of patients who received involuntary ECT. ECT remains an important aspect of mental health care in Australia, particularly in the public mental health services which provide care for many individuals with severe treatment refractory psychosis. More studies are needed to generate a greater understanding of the factors influencing the provision of ECT within differing geographical, social and healthcare landscapes.

## Supplemental Material

sj-docx-1-anp-10.1177_00048674241256839 – Supplemental material for Electroconvulsive therapy in a tertiary Australian mental health facility between 2009 and 2020 Supplemental material, sj-docx-1-anp-10.1177_00048674241256839 for Electroconvulsive therapy in a tertiary Australian mental health facility between 2009 and 2020 by Emily Martin, Subramanian Purushothaman, Emma Ballard, Julie A Blake, Kylie Burke and James G Scott in Australian & New Zealand Journal of Psychiatry
