# Supplementary material for: Electroconvulsive therapy in a tertiary Australian mental health facility between 2009 and 2020
Source: Aust N Z J Psychiatry. 2024 Jun 3;58(8):713–20. doi: 10.1177/00048674241256839 (PMC11892079; doi:10.1177/00048674241256839)
Supplement: sj-docx-1-anp-10.1177_00048674241256839 – Supplemental material for Electroconvulsive therapy in a tertiary Australian mental health facility between 2009 and 2020 [file sj-docx-1-anp-10.1177_00048674241256839.docx]

**Supplementary Table 1:** Summary of acute electroconvulsive therapy by diagnosis.

| **Treatment** | **Overall** | **Schizophrenia** | **Schizoaffective disorder** | **Psychotic disorder** | **Bipolar** | **Depression** | **Other** |
| --- | --- | --- | --- | --- | --- | --- | --- |
|  | n (%) | n (%) | n (%) | n (%) | n (%) | n (%) | n (%) |
| **Number of patients (n)** | 667 | 247 | 77 | 17 | 90 | 225 | 11 |
| **Number of courses (n)** | 1159 | 441 | 162 | 26 | 174 | 342 | 14 |
| **Number of treatments (n)** | 8543 | 3547 | 1185 | 210 | 1194 | 2329 | 78 |
| **Gender (male)** | 365 (54.7) | 173 (70.0) | 39 (50.6) | 10 (58.8) | 40 (44.4) | 98 (43.6) | 5 (45.5) |
| **Age range at time of treatment** |  |  |  |  |  |  |  |
| <18 | 266 (3.1) | 34 (1.0) | 14 (1.2) | 19 (9.0) | 36 (3.0) | 156 (6.7) | 7 (9.0) |
| 18 to 24 | 625 (7.3) | 291 (8.2) | 25 (2.1) | 26 (12.4) | 171 (14.3) | 98 (4.2) | 14 (17.9) |
| 25 to 29 | 637 (7.5) | 283 (8.0) | 60 (5.1) | 36 (17.1) | 70 (5.9) | 188 (8.1) | 0 (0.0) |
| 30 to 34 | 966 (11.3) | 473 (13.3) | 91 (7.7) | 59 (28.1) | 165 (13.8) | 157 (6.7) | 21 (26.9) |
| 35 to 49 | 2537 (29.7) | 1352 (38.1) | 368 (31.1) | 54 (25.7) | 350 (29.3) | 405 (17.4) | 8 (10.3) |
| 50 to 64 | 2175 (25.5) | 853 (24.0) | 513 (43.3) | 0 (0.0) | 228 (19.1) | 562 (24.1) | 19 (24.4) |
| 65 to 74 | 692 (8.1) | 238 (6.7) | 53 (4.5) | 0 (0.0) | 113 (9.5) | 288 (12.4) | 0 (0.0) |
| >75 | 645 (7.6) | 23 (0.6) | 61 (5.1) | 16 (7.6) | 61 (5.1) | 475 (20.4) | 9 (11.5) |
| **Consent type per course** |  |  |  |  |  |  |  |
| Emergency consent | 240 (20.7) | 87 (19.7) | 40 (24.7) | 13 (50.0) | 50 (28.7) | 45 (13.2) | 5 (35.7) |
| Mental Health review tribunal | 593 (51.2) | 303 (68.7) | 87 (53.7) | 13 (50.0) | 80 (46.0) | 105 (30.7) | 5 (35.7) |
| Voluntary | 326 (28.1) | 51 (11.6) | 35 (21.6) | 0 (0.0) | 44 (25.3) | 192 (56.1) | 4 (28.6) |
| **Course type** |  |  |  |  |  |  |  |
| Community | 37 (3.2) | 8 (1.8) | 5 (3.1) | 0 (0.0) | 3 (1.7) | 20 (5.8) | 1 (7.1) |
| Inpatient | 1122 (96.8) | 433 (98.2) | 157 (96.9) | 26 (100.0) | 171 (98.3) | 322 (94.2) | 13 (92.9) |
| **Median number of courses per patient (median (IQR))** | 1 (1 - 2) | 1 (1 - 2) | 1 (1 - 2) | 1 (1 - 1) | 1 (1 - 2) | 1 (1 - 2) | 1 (1 - 2) |
| **Median number of treatments per patient (median (IQR))** | 9 (6 - 14) | 10 (6 - 16) | 10 (6 - 18) | 8 (6 - 11) | 8 (6 - 15) | 8 (5 - 12) | 7 (6 - 9) |
| **Median days between treatments per patient (median (IQR))** | 2 (2 - 3) | 3 (2 - 3) | 2 (2 - 3) | 2 (2 - 3) | 2 (2 - 3) | 2 (2 - 3) | 3 (2 - 3) |

**Supplementary Table 2:** Summary of maintenance electroconvulsive therapy by diagnosis.

|  | **Overall** | **Schizophrenia** | **Schizoaffective disorder** | **Psychotic disorder** | **Bipolar** | **Depression** |
| --- | --- | --- | --- | --- | --- | --- |
|  | n (%) | n (%) | n (%) | n (%) | n (%) | n (%) |
| **Number of patients (n)** | 97 | 52 | 13 | 1 | 7 | 24 |
| **Number of courses (n)** | 510 | 319 | 45 | 3 | 37 | 106 |
| **Number of treatments (n)** | 4417 | 3208 | 310 | 12 | 325 | 562 |
| **Gender (male)** | 54 (55.7) | 38 (73.1) | 5 (38.5) | 0 (0.0) | 1 (14.3) | 10 (41.7) |
| **Age range at time of treatment** |  |  |  |  |  |  |
| <18 | 16 (0.4) | 0 (0.0) | 16 (5.2) | 0 (0.0) | 0 (0.0) | 0 (0.0) |
| 18 to 24 | 13 (0.3) | 1 (0.0) | 0 (0.0) | 0 (0.0) | 0 (0.0) | 12 (2.1) |
| 25 to 29 | 53 (1.2) | 52 (1.6) | 0 (0.0) | 0 (0.0) | 0 (0.0) | 1 (0.2) |
| 30 to 34 | 447 (10.1) | 377 (11.8) | 0 (0.0) | 0 (0.0) | 28 (8.6) | 42 (7.5) |
| 35 to 49 | 1705 (38.6) | 1475 (46.0) | 78 (25.2) | 0 (0.0) | 7 (2.2) | 145 (25.8) |
| 50 to 64 | 1541 (34.9) | 1200 (37.4) | 214 (69.0) | 0 (0.0) | 10 (3.1) | 117 (20.8) |
| 65 to 74 | 292 (6.6) | 103 (3.2) | 0 (0.0) | 0 (0.0) | 153 (47.1) | 36 (6.4) |
| >75 | 350 (7.9) | 0 (0.0) | 2 (0.6) | 12 (100.0) | 127 (39.1) | 209 (37.2) |
| **Consent type per course** |  |  |  |  |  |  |
| Emergency consent | 4 (0.8) | 1 (0.3) | 1 (2.2) | 0 (0.0) | 1 (2.7) | 1 (0.9) |
| Mental Health review tribunal | 267 (52.4) | 198 (62.1) | 18 (40.0) | 1 (33.3) | 33 (89.2) | 17 (16.0) |
| Voluntary | 239 (46.9) | 120 (37.6) | 26 (57.8) | 2 (66.7) | 3 (8.1) | 88 (83.0) |
| **Course type** |  |  |  |  |  |  |
| Community | 438 (85.9) | 259 (81.2) | 40 (88.9) | 3 (100.0) | 32 (86.5) | 104 (98.1) |
| Inpatient | 72 (14.1) | 60 (18.8) | 5 (11.1) | 0 (0.0) | 5 (13.5) | 2 (1.9) |
| **Median number of courses per patient (median (IQR))** | 2 (1 - 2) | 3 (1 - 9) | 2 (1 - 5) | 3 (na) | 4 (1 - 8) | 3 (1 - 5) |
| **Median number of treatments per patient (median (IQR))** | 16 (9 - 56) | 28 (9 - 74) | 15 (5 - 42) | 12 (na) | 17 (10 - 127) | 14 (7 - 32) |
| **Median days between treatments per patient (median (IQR))** | 11 (7 - 14) | 7 (7 - 14) | 14 (9 - 14) | 14 (na) | 14 (7 - 21) | 14 (4 - 14) |

na – not applicable
